# Bioluminescence-based visualization of CD4 T cell dynamics using a T lineage-specific luciferase transgenic model^1^

**DOI:** 10.1186/1471-2172-10-44

**Published:** 2009-08-03

**Authors:** Joseph H Chewning, Kari J Dugger, Tandra R Chaudhuri, Kurt R Zinn, Casey T Weaver

**Affiliations:** 1 Department of Pediatrics, University of Alabama at Birmingham, Birmingham, Alabama, USA; 2 Department of Microbiology, University of Alabama at Birmingham, Birmingham, Alabama, USA; 3 Radiology Department, University of Alabama at Birmingham, Birmingham, Alabama, USA; 4 Department of Medicine, University of Alabama at Birmingham, Birmingham, Alabama, USA; 5 Department of Pathology, University of Alabama at Birmingham, BBRB 870, 845 19th St. S, Birmingham, Alabama, 35294, USA

## Abstract

**Background:**

Rapid clonal expansion of T cells occurs in response to antigenic challenges. The kinetics of the T cell response has previously been described using tissue-based studies performed at defined time points. Luciferase bioluminescence has recently been utilized for non-invasive analysis of *in vivo *biologic processes in real-time.

**Results:**

We have created a novel transgenic mouse model (T-Lux) using a human CD2 mini-gene to direct luciferase expression specifically to the T cell compartment. T-Lux T cells demonstrated normal homing patterns within the intact mouse and following adoptive transfer. Bioluminescent signal correlated with T cell numbers in the whole body images as well as within specific organ regions of interest. Following transfer into lymphopenic (RAG2^-/-^) recipients, homeostatic proliferation of T-Lux T cells was visualized using bioluminescent imaging. Real-time bioluminescent analysis of CD4^+ ^T cell antigen-specific responses enabled real-time comparison of the kinetics and magnitude of clonal expansion and contraction in the inductive lymph node and tissue site of antigen injection. T cell expansion was dose-dependent despite the presence of supraphysiologic numbers of OVA-specific OT-II transgenic TCR T-Lux T cells. CD4^+ ^T cells subsequently underwent a rapid (3–4 day) contraction phase in the draining lymph node, with a delayed contraction in the antigen delivery site, with bioluminescent signal diminished below initial levels, representing TCR clonal frequency control.

**Conclusion:**

The T-Lux mouse provides a novel, efficient model for tracking *in vivo *aspects of the CD4^+ ^T cell response to antigen, providing an attractive approach for studies directed at immunotherapy or vaccine design.

## Background

T cell migration to sites of inflammation and infection is essential for adaptive immunity and host protection. Current methods of analyzing T cell migration and homing primarily rely on obtaining mouse end-organ tissues and subsequent detection of cells within these organs, either through in situ analysis by immunohistologic methods or by isolation and analysis of recovered T cells from disrupted tissues. While each of these approached offers unique advantages (and disadvantages), neither permits "real-time" analysis of T cell dynamics following antigenic exposure and is limited to specific time points and tissues chosen for study. Bioluminescent imaging represents a powerful alternative for the monitoring immune cell homing and migration *in vivo *that does not require animal sacrifice for analysis.

Luciferase proteins produce light in the visible spectrum (approximately 560 nm for firefly luciferase) following interaction with luciferin substrate molecules. This reaction only requires ATP and oxygen and thus can occur in any actively metabolic cell. Luciferin, a small water-soluble molecule, readily crosses cell membranes and can penetrate into virtually all tissues. Light produced by the luciferase-luciferin reaction is detectable by low-light detection devices, such as charge-coupled device (CCD)^4^cameras. Bioluminescence imaging is a good model for *in vivo *imaging based on the low background signal, since it only detects chemical reactions between the enzyme and substrate. Bioluminescence also requires relatively short imaging times (seconds to minutes), is easy to use, and the instrumentation is relatively inexpensive [1,2].

Luciferase imaging techniques have previously been used to track localization of bacterial and viral pathogens, monitor therapeutic responses in tumor xenografts, and allograft cell survival [3-6]. Recent studies have utilized bioluminescence to track T cell homing patterns following adoptive transfer of luciferase-expressing allogeneic T cells into MHC-mismatched recipient mice [7,8]. We have also reported use of luciferase-expressing CD8 T cells for analysis of population dynamics in response to viral-based antigenic-challenge [9]. These studies reveal that T cell proliferation, in the context of graft versus host disease or viral infection, can be visualized in 'real-time' within recipient mice. The luciferase-expressing T cells can also be identified within specific tissue sites in order to determine homing and migration kinetics over time [7,8].

In the current study, we describe the generation of a transgenic mouse (T-Lux) model in which the luciferase gene is specifically expressed by T cells, thereby permitting analysis of T cell population dynamics in living mice in real-time. By crossing the T-lux transgenic mice with OVA-specific CD4 TCR transgenic mice (OT-II), we have generated a reporter model with which to track antigen-specific CD4 T cells *in vivo*, in real-time. Our studies reveal the population dynamics of the CD4 clonal response within draining lymphoid tissues and antigen injection sites in individual mice and demonstrate the utility of this model for non-invasive analyses of T cell clonal responses.

## Methods

### Mice

C57BL/6 and C57BL/6.Ly5.2 (CD45.1^+^) mice were obtained from Jackson Laboratory (Bar Harbor, ME). Thy1.1-expressing C57BL/6 mice were obtained from Charles River Breeding Laboratories. Mice used for imaging were placed on a low light diet (Harlan Tekland) to reduce non-specific luminescence. All mice were housed or bred in our specific pathogen-free facility, and were initially used at 6–10 weeks of age. All mice were housed and treated according to National Institutes of Health guidelines under the auspices of the UAB Institutional Animal Care and Use Committee (IACUC) of the University of Alabama at Birmingham.

### Generation of T-lux transgenic mouse

The luciferase coding sequence was excised from the pGL3 plasmid (Promega) by Xba I/Nco I digestion, blunted with Klenow and ligated into a Sma I-linearized hCD2 minigene cassette plasmid (the kind gift of Dr. Dmitri Kioussis; [10]). The T-lux transgene was restriction mapped for correct orientation and sequenced across the integration joints to confirm the correct reading frame and terminus of the luciferase gene (using primers OCW938 and OCW939). The reporter gene construct was introduced into single-cell embryos of donor C57BL/6 mice by pronuclear injection in the UAB Transgenic Mouse Facility. Potential founder lines were screened by luminescence analysis of peripheral blood. Red blood cells were lysed from 30 μL of blood using ACK buffer for 2 minutes. Cells were then plated into 96-well plates and luciferin (100 μg/ml) added to each well 10 minutes prior to imaging. Bioluminescence imaging of the plates was performed using the IVIS^® ^Imaging System with stage at 15 cm height for 600 seconds. One founder line (T-lux 9) with readily detectable luminescence that transmitted the transgene in the germline was selected for subsequent studies.

### Antibodies

The following antibodies were used for fluorescence labelling of cells for flow cytometric analyses: fluorescein isothiocyanate (FITC)-labelled anti-mouse CD8 (clone 53-6.7); phycoerythrin (PE)-labelled anti-mouse CD4 (RM4-5); PE-labelled anti-mouse CD45.2 (104); biotin-labelled anti-mouse CD8 (H35-17.2) and CD45.1 (A20). All were purchased from eBiosciences (San Diego, CA). PerCP-labelled anti-mouse CD3 (145-2C11) was purchased from BD Pharmingen (San Jose, CA).

### Flow cytometric analysis

Single cell suspensions from specified target organs were analyzed by FACS at the indicated time points. Cells were stained for flow cytometry with FITC-, PE-, PerCP-, or biotin-conjugated mAbs indicated in the appropriate figure legends. Cells labelled with biotinylated primary Ab were detected with allophycocyanin-conjugated streptavidin. For analytical flow cytometry, at least 100,000 events with forward and side scatter properties of lymphocytes were collected on a FACSCalibur flow cytometer (BD Biosciences, San Jose, CA) and analyzed using CellQuest software (BD Biosciences, San Jose, CA). Some analyses were performed using FlowJo software (Treestar, Inc, Ashland, OR).

### Isolation and Transfer of T-lux Cells

Splenocytes were harvested from T-lux mice and CD4^+ ^T lymphocytes isolated using Dynal Bead Separation (Dynal Biotech LLC), according to manufacturer's instructions. Briefly, T-lux splenocytes were incubated with CD4^+ ^specific magnetic beads for 20 minutes at 4°C. The cell-attached beads were then incubated with Detach-a-bead at room temperature for 1 hour. Thoroughly washed cells were counted, resuspended in RPMI medium without serum, and transferred to recipient mice via tail vein injection.

### *In vivo *Bioluminescence Imaging of Mice

Mice were anesthetized with isofluorane gas and placed in a light-tight chamber. A photographic (gray-scale) reference image was obtained at 10 minutes after D-luciferin injection (2.5 mg intraperitoneal); bioluminescent images were collected immediately thereafter. Images were obtained with a CCD camera cooled to -120°C, using the IVIS^® ^100 Imaging System (Xenogen Corp., Alameda, CA) with the field of view set at 10 cm height. The photographic images used a 0.2 second exposure, 8f/stop, 2 binning (resolution), and an open filter. The bioluminescent images used exposures ranging from 120 to 600 seconds, 1f/stop, 8 binning and open filter. The bioluminescent and gray-scale images were overlaid using Living Image software (Xenogen Corp.). Igor image analyses software (Wavemetrics, Lake Oswego, OR) was also used to obtain a pseudocolor image representing bioluminescence intensity (blue, least intense, and red, most intense). Regions of interest were drawn around organs and the total counts (photons) were summed in the entire organ areas. The total counts in each region of interest were normalized to total acquisition time to obtain counts/sec.

## Results

### Transgenic mice containing the firefly luciferase gene, *luc*, under control of a human CD2 mini-gene cassette produce T cell-specific bioluminescence

The human CD2 (hCD2) minigene cassette has previously been utilized to direct transgene expression in mouse T cells [10-12]. The hCD2 minigene contains an ~5 kb 5' flanking/promoter region and a 5.5 kb 3' region that contains a locus control region (LCR). The LCR of the hCD2 contains a T cell-specific enhancer and additional regulatory elements responsible for establishing an active chromatin domain in T lymphocytes [12]. The intact first intron of human CD2, located downstream of the promoter region, improves efficiency of transgene expression. Transgene expression levels are typically independent of the integration site, and the level of expression is proportional to the number of transgene copies [11].

**Figure 1:**
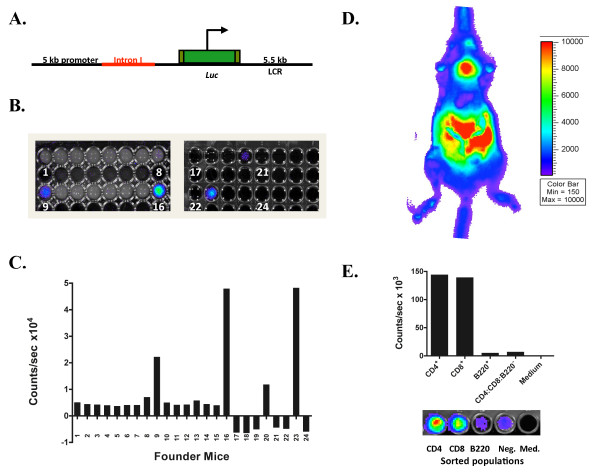
**T-lux Transgenic Mouse Model**. Shown in (A) is a diagrammatic representation of the CD2.luc transgene. The *luc *gene is located downstream of the 5 kb promoter region and the intact first intron (in red). The CD2.luc transgene was introduced into single-cell embryos of C57Bl/6 mice by standard transgenesis. Equal numbers of peripheral blood mononuclear cells from potential founder lines were screened for luciferase activity in 96 well plates (B). Numbers in positive wells indicate founder line. Actual light emission results are shown in (C), confirming positivity of founder lines #9, 16, 20, and 23. (D) Whole-body bioluminescence image of a transgenic 'T-lux' mouse. A strong primary lymphoid tissue bioluminescent signal (thymus) and secondary lymphoid tissue signal (spleen, mesenteric lymph nodes (LNs), and peripheral LNs) is seen. Pseudocolor scale is shown to right of image. Lineage specificity of transgene expression was confirmed by FACS sorting of splenocytes into four populations, each containing 1 × 10^6 ^cells (E). These populations were: CD4^+ ^T cells, CD8^+ ^T cells, B220^+ ^(B cells), and CD4^-^CD8^-^B220^- ^(non-T, non-B cells). A representative bioluminescent image of wells containing each sorted cell population is shown below figure.

Firefly luciferase gene, *luc*, was inserted into the partial second exon of hCD2 minigene (Figure 1A). The transgene was then injected into the pronuclei of single-cell embryos from C57BL/6 female mice. Two rounds of injections resulted in 24 mice. Red blood cell-depleted peripheral blood was imaged after the addition of luciferin, using the IVIS^® ^imaging system. Four founder mice that displayed luciferase expression were identified (T-lux founder lines 9, 16, 20, and 23) (Figure 1B and 1C). One of these founders, T-lux founder #9, transmitted the transgene in the germline and displayed T lineage-specific expression (below), and was used for all subsequent studies.

The distribution of luciferase-expressing cells was determined in intact founder mice. Bioluminescent imaging revealed increased signal within lymphoid tissue regions (Figure 1D). As anticipated, bioluminescence was detected from the thymus, as well as spleen, peripheral lymph nodes (LNs), and gastrointestinal lymphoid tissues, consistent with normal lymphocyte homing patterns [13-15]. Light emission from circulating T cells and skin T cells delineated the anatomical outlines of the mouse.

Splenocytes from T-lux transgenic mice were isolated in order to determine the lineage specificity of the luciferase transgene. Splenocytes were separated by FACS into four populations, CD4^+^, CD8^+^, B220^+^, and a triple-negative population. The FACS purity of the sorted populations of CD4^+ ^and CD8^+ ^were 98% and 99%, respectively, while the B220^+ ^and the negative populations were 96% and 90% pure. Cells were plated into 96-well plates in equivalent numbers of each population (1 × 10^6 ^cells/well) and were imaged for bioluminescence *ex vivo*. As shown in Figure 1E, bioluminescent signal was almost exclusively within the CD4^+ ^and CD8^+ ^T cell populations. Quantification of light emission of these cell populations confirmed the purity of the sort. Ratios of CD4:B220 and CD4:negative show a 4% and 5% bioluminescent signal, respectively. These numbers are consistent with the low level of T cell contamination of these populations detected by FACS analysis. T-lux T cells retained normal T cell proliferation in response to TCR stimulation and produced similar levels of cytokines compared to wild-type T cells (data not shown). Thus, the T-lux transgene produces a detectable bioluminescent signal that is limited to the T cell compartment, and does not alter T cell function in these cells.

### T-lux CD3^+ ^T cells rapidly home to secondary lymphoid tissues and produce a stable luciferase signal within 24 hours following adoptive transfer

Naïve T cells are excluded from non-lymphoid tissues and preferentially home to secondary lymphoid organs [16]. Expression of CD62L and CCR7 allow for recognition and binding to the high endothelial venules within lymphoid tissues [17,18]. T cells typically reside within the lymph nodes for approximately 24 hours before re-entering the circulation unless activated by dendritic cells [19,20]. Until antigen is encountered, these T cells will migrate randomly between secondary lymphoid tissues [21].

**Figure 2:**
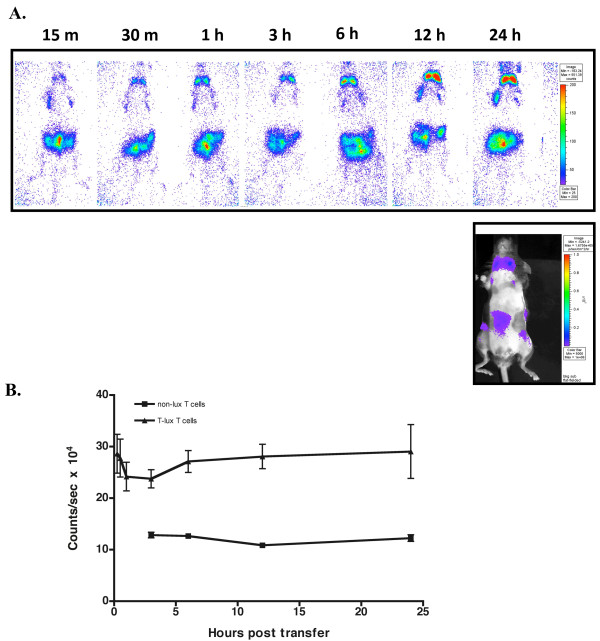
**Kinetics of Tissue Localization following Adoptive Transfer of T-lux T cells**. The kinetics of naïve T cell homing were assessed following adoptive transfer of 1 × 10^7 ^T-lux CD3^+ ^cells into CD45.1-positive, C57BL/6 recipients (N = 8). Recipient mice were imaged for whole body bioluminescence at timed intervals following transfer. (A) Representative images from a single mouse for each of the indicated time points. Additional reference anatomical map shown below 24-hour time point. Pseudocolor scales are shown to right of figures. The mean bioluminescent signal of the cohort for each time point is displayed in (B). Control bioluminescence from mice receiving non-transgenic T cells ('non-lux' T cells) is also shown. Error bars indicate SEM.

The ability to monitor T cell signal within the live mouse over time provides an opportunity to efficiently analyze T cell distribution kinetics serially in single mice, eliminating the need for use of large cohorts of mice that can be analyzed only at individual time points. To test the homing characteristics of the T-lux T cells following adoptive transfer into immunocompetent mice, we transferred 1 × 10^7 ^T-lux CD3^+ ^cells into CD45.1, non-irradiated B6 recipients (n = 8). Individual recipient mice were imaged beginning 15 minutes post-transfer and at the indicated time intervals (Figure 2A). Detectable signal was present within the peripheral and mesenteric lymph nodes as early as 15 min following adoptive transfer, consistent with previously published studies [20]. A splenic signal was also visible prior to 1 hour after transfer in anterior-posterior (AP) image orientations, and was readily detectable 15 min post-transfer in left lateral images (not shown).

Bioluminescent signal (counts/sec) was calculated in order to quantitate the bioluminescent signal intensity change over time, as T cell migratory patterns can be delineated by analyzing the signal flux within different regions of measurement. When the signal flux ceases to change, it can be concluded that pool size homeostasis has been achieved in the anatomical regions of interest. We collected and graphed whole body light emission measurements over time and assessed migratory trends of T-lux T cells. Following a brief decrease in signal due to redistribution following injection, whole body bioluminescence increased, reaching a plateau between 12 and 24 hours (Figure 2B). Notably, signal intensity in the intestinal lymphoid tissues equilibrated more rapidly (1–3 hours post-transfer), consistent with more rapid recruitment to these tissue sites prior to redistribution, whereas signal intensities in other secondary lymphoid tissues equilibrated by ~12 hours post-transfer (Figure 2A, and data not shown). There was no detectable early enrichment in the lung fields, indicating that no significant trapping of transferred T cells in the pulmonary capillary beds was evident.

### Correlation of bioluminescent signal intensity with T cell distribution *in vivo*

**Figure 3:**
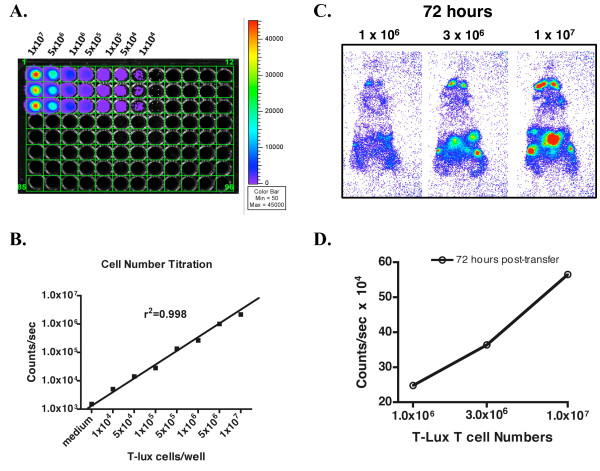
**Correlation of T Cell Dose and Luciferase Bioluminescent Signal**. A comparison of T-lux T cell numbers with light emission signal was done both *in vitro *and *in vivo *to assess the sensitivity of the T cell luciferase signal. (A) *Pooled *CD3^+ ^T cells from spleen and peripheral lymph nodes of T-lux mice were plated in triplicate (*in columns*) into a 96-well plate at the indicated cell numbers per well, and light emission was quantitated following addition of luciferin. Pseudocolor scale is shown to right. (B) Plot of light emission data from (A) versus input T cell numbers. Error bars indicate standard error of the mean (SEM). Medium alone was used as negative control. The r^2 ^value for data is displayed on figure (see text for details). (C) CD3^+ ^T cells isolated from pooled spleen and LN of T-lux mice were adoptively transferred into congenic C57BL/6 recipients. Whole body bioluminescence was quantified at 12, 24, and 72 hours post-transfer. Representative images from 72 hours post-transfer are shown for three cell doses. (D) Whole body luminescence was plotted against transferred T cell numbers in (C). A representative plot is shown for the 72-hour time point.

To determine the relationship between bioluminescent signal and T cell numbers in the T-lux mouse, the per-cell signal intensity was defined under non-attenuating conditions *in vitro *to provide a baseline for subsequent *in vivo *studies. *In vitro*, T-lux spleen and peripheral lymph node T cells were added to a 96-well plate in decreasing cell numbers from 1 × 10^7 ^to 1 × 10^4 ^CD3^+ ^cells. Bioluminescent signal was quantified for each population (Figure 3A). A comparison of signal strength (counts/sec) and T cell numbers demonstrated a linear relationship (r^2 ^= 0.998) (Figure 3B), and the per-cell signal intensity was ≈ 0.225 counts/cell/sec.

The signal of T cell bioluminescence within the intact mouse was assessed following adoptive transfer of luciferase-expressing cells. Initial studies were designed to analyze whole body bioluminescent signal generated by differing numbers of T-lux T cells. Increasing numbers of CD3^+ ^T-lux cells (1 × 10^6^, 3 × 10^6^, and 1 × 10^7^; n = 3) were injected via tail vein into congenic C57BL/6 recipients. Bioluminescent imaging of the entire mouse was performed at 12, 24, and 72 hours following transfer. Representative images obtained at 72 hours are shown in Figure 3C. Stable bioluminescent signals in the abdominal region, spleen, and cervical lymph node regions were present by 12 hours post-transfer (data not shown). Comparison of whole body counts/sec versus T-lux cell dose confirmed a linear and proportional relationship (Figure 3D).

**Figure 4:**
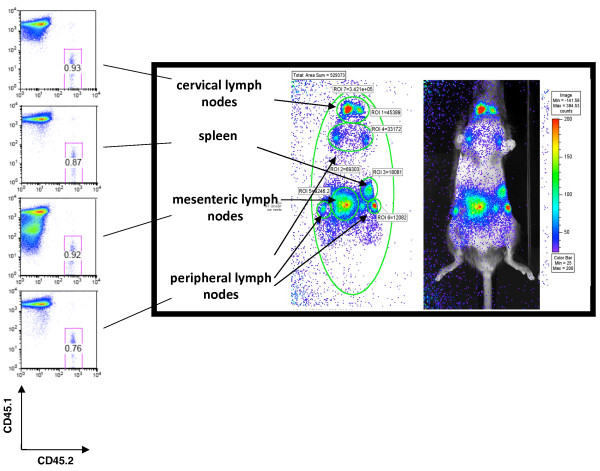
***In vivo *Bioluminescent Signal Within Different Lymphoid Tissues**. To determine the correlation between T-lux T cell numbers and bioluminescent signal *in vivo*, we adoptively transferred T-lux CD3^+ ^cells (3.5 × 10^6^) into CD45.1-positive, congenic B6 recipients. Three days post-transfer, light emission was determined for whole body, as well as defined anatomical regions (Regions of Interest, or ROI), including cervical, peripheral, and mesenteric lymph nodes and spleen, and compared to actual T cell numbers recovered from these tissues. FACS analysis was performed on cells obtained from recipient mice and representative plots are shown (left panel). Tissue source is indicated for each FACS plot. Cells were first gated on live CD3-positive lymphocytes. Recipient CD45.1 allotype is shown on the y-axis and donor CD45.2 on x-axis. Numbers indicate the percentages of donor (T-lux) T cells of the total live CD3+ T cells in each tissue. The composite of bioluminescence images (right panel; left image) shows a non-normalized pseudocolor image, with the analyzed ROIs indicated by arrows; the same pseudocolor image is overlayed on a photographic image of the mouse (right panel; right image).

Subsequent experiments were performed to determine the correlation of T cell number to bioluminescent signal within specific anatomical tissue regions. T cells (3.5 × 10^6^) were obtained from CD45.2^+ ^T-lux mice and introduced into congenic CD45.1^+ ^C57BL/6 recipients. Use of the CD45 allelic marker allowed discrimination by FACS of the donor CD45.2-expressing T-lux cells. Recipient mice were imaged every three days in defined anatomical regions following T cell redistribution to secondary lymphoid tissues. Direct analysis of transgenic T cell numbers in those sites by flow cytometry was then performed and compared to the luciferase bioluminescent signal (Figure 4).

The peripheral lymph node (inguinal and axillary) regions of interest (ROIs) generated an observable signal following T cell transfer (3.5 × 10^6^) at all time points (Figure 4). Comparison of signal strength to T cell numbers resulted in a 1.54 counts/cell/sec ratio (Table 1). Cervical lymph nodes produced similar results of 1.06 counts/cell/sec. By comparison, the signal to cell ratio within the spleen was reduced in AP positioning (0.075 counts/cell/sec), consistent with increased attenuation of luciferase signal from T cells resident in the deeper tissues (Table 1), although on lateral view, this attenuation was not apparent (data not shown).

**Table 1:** Detection Efficiency of T-Lux T cells

Region of Interest (ROI)	Mean counts/cell/sec (Stdev^a^)
Cervical lymph nodes	1.06 (0.14)
Mesenteric lymph nodes	1.54 (0.20)
Peripheral lymph nodes	2.04 (0.25)
Spleen^b^	0.075 (0.015)

^a ^Stdev, standard deviation

^b ^Anterior-posterior (AP) imaging view

Signal to cell ratios from mesenteric lymph nodes surprisingly revealed an increased signal to cell ratio (2.04 counts/cell/sec) compared to superficial lymph nodes (Figure 4 and Table 1). This is likely due to a contribution of additional signal from other lymphoid tissues within the gut, producing a "generalized" signal. Additional experiments were performed on mice after surgically opening the abdomen under anesthesia and exposing intestinal tissue. These "open" images revealed T-lux signal within individual Peyer's patches and other regions not clearly seen within the overall abdominal signal (data not shown). Collectively, these experiments demonstrated the ability to quantify T cell numbers *in situ *in real-time using bioluminescence, and although the signal intensity per cell varied depending on anatomic location, the calculated values proved reliable for estimating T cell number in each site at different time points in the same animal, and even between individual mice that received comparable T cell transfers.

### Tracking homeostatic proliferation in lymphopenic hosts

Normal T cells undergo extensive homeostatic proliferation in a lymphopenic setting. Expansion of T cells following transfer into an immunodeficient host can be due to both thymic-dependent and -independent mechanisms [22]. An essential role for interleukin-7 in thymic-independent expansion of T cells has been well described [23,24]. The luciferase-expressing T-lux mouse provided a model to visualize homeostatic T cell expansion over time within the live host.

**Figure 5:**
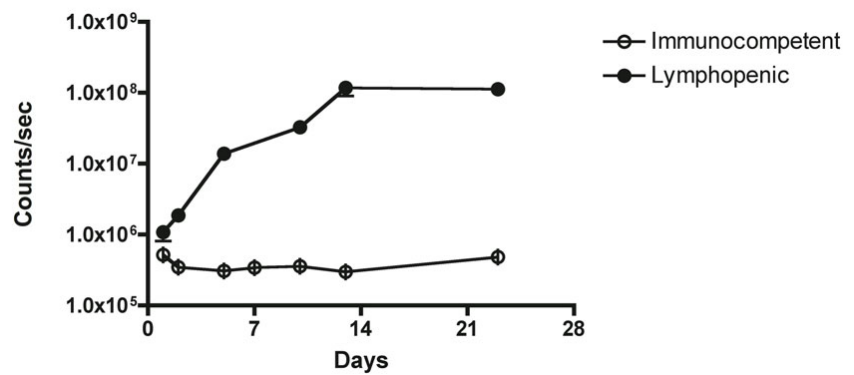
**Homeostatic Proliferation of T-lux Cells**. T-lux CD3^+ ^T cells (1 × 10^7^) were transferred to WT, congenic C57BL/6 (B6) mice (N = 8), denoted 'immunocompetent,' or RAG-deficient (RAG^-/-^) B6 mice (N = 4), denoted 'lymphopenic.' Whole body bioluminescent imaging was performed at the indicated intervals post-transfer for a total of 24 days, and light emission was determined for both groups. Error bars indicate SEM.

We transferred 1 × 10^7 ^T-lux CD3^+ ^cells into C57BL/6 RAG-deficient (RAG^-/-^) mice (n = 4), which lack mature T and B cells. Reconstituted RAG^-/- ^mice were then imaged for bioluminescent signal at the indicated time points post-transfer. Whole body bioluminescent images revealed rapidly increasing signal during the first two weeks following transfer (~100-fold increase), followed by plateau. Comparison studies performed with transfers of 1 × 10^7 ^T-lux CD3^+ ^cells into WT C57BL/6 mice showed that, in contrast to the luciferase signal in the lymphopenic RAG^-/- ^mice, immunocompetent mice showed a modest decline in luciferase signal after two days, followed by a relatively stable signal over the ensuing three weeks (Figure 5). In the RAG-deficient recipients, the T cell signal was evident in both lymphoid and non-lymphoid tissues, consistent with some degree of memory T cell development and distribution. Whole body bioluminescence signal from reconstituted RAG-/- mice was approximately 20% of the intact, wild-type T-lux mouse. This finding correlated with the relative total T cell numbers when organs were harvested and transferred cells counted (data not shown).

### Non-invasive tracking of T cell half-life

Diversity of the T cell receptor repertoire is essential for protection of the host from infection. Previous studies have demonstrated that the number of cells bearing the same MHCII:peptide-specific TCR within the naïve CD4^+ ^T cell repertoire is in the range of 20–200 [25]. This same group also demonstrated that survival of CD4^+ ^T cells *in vivo *is determined in part by TCR specificity attributes, but also clonal frequency, with an inverse relationship between T cell number and half-life. The half-life of adoptively transferred polyclonal CD4^+ ^T cells within secondary lymphoid tissues was calculated to be 124 days [26].

**Figure 6:**
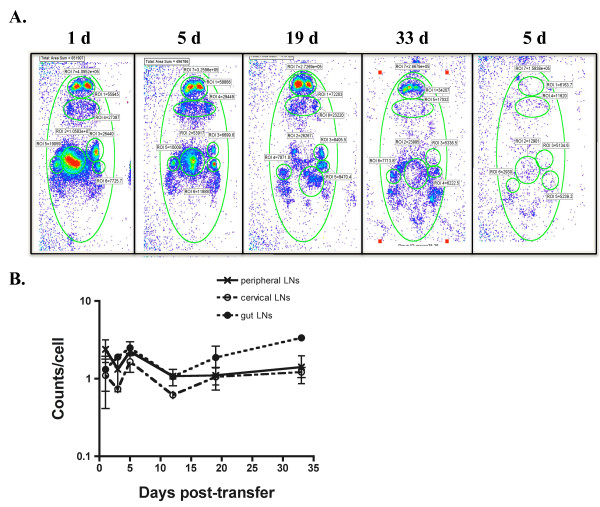
**Half-Life and Signal Stability of Transferred T-lux Cells *In Vivo***. (A) T-lux T cells (3.5 × 10^6 ^cells) were adoptively transferred into congenic C57BL/6 (B6) mice (N = 8), and whole body bioluminescent imaging was obtained at the indicated times post-transfer. Control mice were given non-transgenic T cells; a representative day 5 image obtained from control mouse is shown to far right. (B) 3.5 × 10^6 ^T cells from CD45.2^+ ^T-lux mice were adoptively transferred into congenic, CD45.1^+ ^B6 mice and tissue-specific light emission quantitated. Light emission was calculated at the indicated times post-transfer for specific regions of interest (ROIs), including peripheral, cervical, and mesenteric (gut) lymph nodes. T-lux T cell numbers from recovered tissues were determined by flow cytometric quantitaton of CD45.2^+ ^cells, and counts/sec per T cell were calculated. T-lux bioluminescent emission (counts/cell) within the indicated regions of interest is plotted over time. Error bars indicate SEM.

Whole body bioluminescent imaging using T-lux T cells provided a non-invasive method of measuring the half-life of T cells within the entire mouse. Adoptive transfer studies were performed by infusing 3.5 × 10^6 ^polyclonal naïve T-lux CD3^+ ^cells (CD45.2-positive) into CD45.1-positive, B6 mice. Bioluminescent imaging was performed on recipient mice every other day, beginning 1 day post-transfer and for a total of 33 days, and in some studies extended for a total of 120 days. As seen in Figure 6A, there was a progressive decline in bioluminescent signal from individual mice following transfers. In extended experiments, the T cell half-life calculated on the basis of decay in luminescent signal yielded a calculated half-life of ~120 days, in good agreement with published data [26]. Further, the decline in signal did not appear to be due to active immune clearance, as parallel studies using non-transgenic, CD45.2-positive, CD3+ T cells transferred into CD45.1 B6 recipients demonstrated a comparable decline in T cell numbers as determined by FACS analysis of lymphoid tissues (data not shown).

We next determined whether there was stability of single-cell luciferase signal by T-lux cells following adoptive transfers. After transfer of 3.5 × 10^6 ^T-lux CD3^+ ^cells, we imaged distinct regions of interest (ROIs) for peripheral, cervical, and mesenteric lymph nodes. Spleen was not included in this analysis due to the attenuation of signal previously demonstrated in AP determinations. We obtained bioluminescent signal measurements from all ROIs every 3 days for 5 weeks. We then quantified T cell numbers from each region using flow cytometric analyses at the indicated time points post-transfer based on the CD45.2 marker, and calculated the counts per cell produced within these lymphoid organs (Figure 6B). The ratio of counts/cell remained constant despite the decline in overall transferred cell numbers, indicating that signal loss was due to decline in the number, not single-cell intensity, of luciferase-expressing cells. Thus, the luciferase reporter appears to be stable for prolonged periods following adoptive transfer and shows no evidence of provoking an alloantigenic response, at least in the C57BL/6 strain background.

### Whole body imaging of clonal CD4+ T cell response to antigen

**Figure 7:**
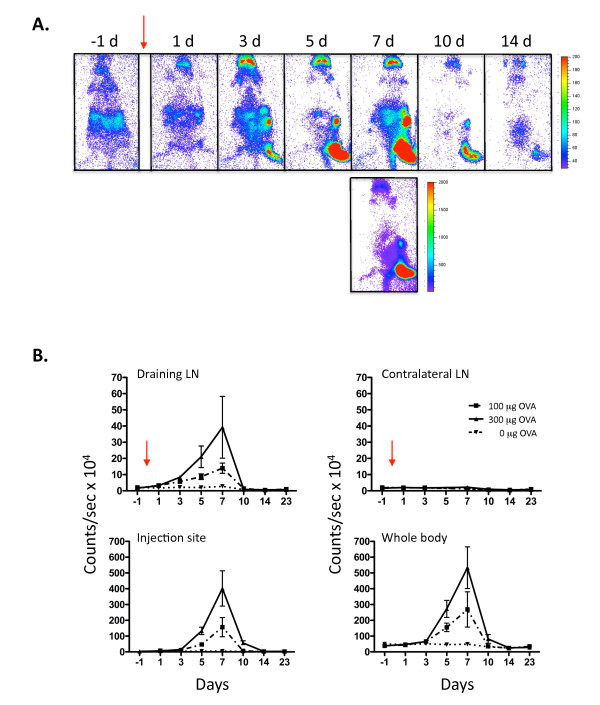
**Clonal T cell Response to Antigen *in vivo***. Naïve CD4+ T cells were isolated from OT-II.T-lux transgenic mice (5 × 10^6^) and transferred into congenic, WT B6 mice on day -2. Following baseline bioluminescent imaging on day -1, mice were given footpad injections of 100 μg or 300 μg of OVA peptide in ME adjuvant (red arrows; day 0). Control mice were given sham footpad injections containing adjuvant only (indicated as 0 μg OVA). Bioluminescence imaging was performed on all mice at the indicated time points. Representative images are shown for mice receiving 300 μg OVA peptide (A). The time of acquisition is shown above individual images ("d" indicates days). Bioluminescence emission (counts/sec) was quantified for all OVA doses and analyzed over time for various regions of interest. Shown in (B) are draining inguinal lymph node, non-draining inguinal lymph node, footpad injection site, and whole body. Error bars indicate SEM.

After encountering antigen, CD4^+ ^T cells undergo marked clonal expansion followed by a contraction phase on clearance of antigen [27]. The T-lux mouse represents a novel model for the analysis of T cell dynamics following antigen encounter. To study the CD4^+ ^T cell antigen-specific response in individual mice, we crossed the T-lux mouse with the OT-II TCR transgenic mouse [28], and transferred T-lux-OT-II T cells into immunocompetent congenic recipients. OVA peptide emulsified in a non-toxic, multiple emulsion adjuvant (ME) [29], or adjuvant alone, was injected into the footpad of recipient mice and the clonal CD4^+ ^T cell response imaged over time (Figure 7).

Quantification of the bioluminescent signal was performed for the draining lymph nodes (LNs), non-draining (contralateral) LNs, dorsal injected footpad, as well as whole body and other regions of interest. Strict gating on the injection site (footpad) was performed to isolate injection site signal from the draining popliteal node signal (Additional File 1). Bioluminescent signal was analyzed over time for each of these sites (Figure 7B). Increased T cell signal was first demonstrated in the draining lymph node at day 1 (24 hours after OVA injection). This signal was greater in the group given 300 μg OVA compared to 100 μg (Figure 7B). Increased signal in the draining lymph node preceded T cell migration to the injection site by 1–2 days, with increasing signal at the injection site apparent on day 3. This pattern is consistent with previous studies demonstrating T cell expansion within the draining lymph node followed by migration and accumulation within non-lymphoid tissue sites [20,30,31]. T cell bioluminescent signal in both these regions, as well as whole body, peaked at day 7 and showed similar patterns of decreasing signal by day 10–12, although residual signal was evident in the injection site for several days after attenuation of signal in the draining LN. T cell signal was higher in whole body, draining lymph node, and footpad regions in those mice given 300 μg compared to 100 μg OVA injections (Figure 7B). The non-draining lymph node showed minimal increase in signal (Figure 7B), but notably, other distal LNs demonstrated significant signal increase at the peak of the response (eg, cervical and mesenteric LNs, day 7). T cell migration to non-lymphoid tissues was largely restricted to the injection site, with minimal apparent trafficking to other tissue sites (Figure 7A). Notably, the T cell signal was substantially higher in the injection site than in the draining LNs at the respective peaks of signal intensity, suggesting that T cells activated in the draining LNs rapidly trafficked to the site of antigen/adjuvant injection and accumulated there before declining in number. Given that the large majority of the total body signal was accounted for in the draining LNs and injection site, the rapid decrement in signal after day 7 indicates cell loss, rather than redistribution to other tissue sites. Thus, following the contraction phase, T cell bioluminescent signal diminished to levels comparable to that of the pre-injection levels (day -1) and those of sham (adjuvant only) injected mice.

## Discussion

T cells undergo a complex pattern of clonal proliferation and expansion following primary and secondary antigenic challenges [32-35]. Using luciferase-expressing OT-II T cells, we were able to monitor antigen-specific CD4^+ ^T cell dynamics serially in the same animal. In accord with previous reports, our results demonstrated initial expansion within the draining lymph node followed rapidly by migration and accumulation within a footpad injection site [20,30]. Notably, and in agreement with a previous study [30], we find that overwhelmingly the T cells that leave the lymph node draining the site of antigen injection migrate to the antigen injection site, although some enhanced accumulation of cells in other secondary lymphoid tissues is evident. Despite early increases in T cells numbers in the draining lymph nodes, T cell numbers within both the draining lymph node and the injection site demonstrated a peak 7 days after injection, followed by a rapid decline in signal intensity both in the draining lymph nodes and injection site that was only marginally delayed kinetically in the latter. The concordance of the peak of T cell expansion in the draining lymph node and injection site in our study is somewhat different from previous studies, which demonstrated a more prolonged time course [20] and kinetic delay between peak T cell numbers in draining lymph node and injection site. This likely reflects the more rapid clearance of the antigen-adjuvant depot in our study, as the ME adjuvant is far less viscous and is more rapidly cleared from tissue sites than the oil-based emulsions used in previous studies (unpublished observations). This highlights the importance of sustained antigen in the injection site to prolong recruitment and/or survival of immigrating T cells attracted to the site of antigen delivery [30].

In the current study, there was no detectable redistribution of CD4 T cell signal to non-lymphoid tissues following clonal contraction in the draining LNs and injection site, which is in contrast to previous studies wherein antigen and adjuvant were delivered systemically, or in studies of the CD8 response to viral infection. Again, this likely reflects differences in the distribution and kinetics of antigen clearance in the different studies. This may also reflect characteristics of the OT-II TCR transgenic model, clonotypic T cells from which have a relatively low avidity for the MHC-OVAp complex that may allow endogenous clones reactive to OVA peptide to out-compete the OT-II response over time. Future studies, following crosses of the T-lux reporter transgene with other TCR transgenic specificities should help address this. In any case, given the relative technical ease with which T cell clonal dynamics can be monitored using the T-lux model, efforts to implement these types of single animal longitudinal studies represents a significant advance for conducting studies of adjuvant type and delivery for future studies, with attendant advantages for optimizing future vaccine therapies for infectious agents and cancer.

Homeostatic proliferation of mature T cells occurs within the lymphopenic host and has been shown to be associated with a concomitant shift toward memory phenotypes (reviewed in [36]). T cell expansion in this setting has been shown to be due to IL-7 [23,24] and IL-15/IL-2-dependent mechanisms [37]. IL-15, in particular, has been demonstrated to induce a memory phenotype in CD8^+ ^T cells that is associated with an alteration of cell homing receptors [38,39]. It is not surprising; therefore, that we observed T cell expansion and localization of T-lux cells within non-lymphoid tissues following transfer into the RAG-deficient mice. Localization within non-lymphoid tissues as well as the activation profile of these cells has important implications in immune reconstitution following infection and bone marrow transplantation, and implementation of the T-lux model for efficient characterization of this process is demonstrated herein.

Bioluminescent imaging technology provides a mechanism to observe T cell homing and specific CD4^+ ^T cell immune responses *in vivo*, in real-time in individual animals. This technique eliminates the need for large groups of mice terminated at varying time points, reducing animal usage and cost. In addition, this provides a method to efficiently observe and quantitate T cell responses in all tissue sites simultaneously, providing a window on T cell response dynamics not easily achieved using approaches that require tissue disruption for localization and quantitation of T cells. Also, given the lack of alloreactivity of the luciferase reporter molecule in the C57BL/6 strain, the T-lux mouse provides an excellent method for long-term immune studies following adoptive transfer. We have monitored T-lux T cell survival up to 75 days following adoptive transfer and found no evidence of immune clearance in re-transfer studies (Figure 6C, and data not shown). Previous studies in rats have demonstrated the absence of immunogenicity of firefly luciferase using transgenic tissue allografts [40]. In contrast, studies using green fluorescence protein (GFP), another bioluminescent tracking molecule, have demonstrated an immune response to the molecule and decreased lifespan for GFP-expressing cells [41]. Thus, the T-lux mouse is ideal for model systems investigating long-term immune recognition, such as mouse bone marrow transplant models. Other groups have utilized luciferase expression for analysis of lymphocyte homing following adoptive transfer [1-4]. These authors have utilized bioluminescence to perform elegant studies on the role of regulatory T cells in graft versus host disease [7,8]. We feel that our T-Lux model improves on previous luciferase models through the generation of T-cell-specific bioluminescence. Limiting luciferase expressing to the T cell compartment enables the specific study of T cell dynamics within the intact mouse and eliminates potential confounding factors, such as an alteration of the normal TCR clonal precursor frequency that can affect T cell dynamics in adoptive transfer studies [26].

A current limitation of the T-lux model is the attenuation of bioluminescent signal from T cells in deeper tissues. This is due to light absorption by superficial tissues situated between the T cell source and the detector when imaging cells within deeper organs. Decreased or attenuated luciferase signal is particularly apparent in the case of interference within blood rich organs, such as spleen, as hemoglobin absorbs light in the wavelength emitted by the luciferase enzyme [42]. However, this limitation only affects absolute quantitation of T cells within deep tissues, while relative quantitation and dynamic changes over time are unaffected, as demonstrated herein. Further, the orientation of the animal can offset much of this limitation, depending on the tissue of interest (e.g., spleen). Finally, recent advances in the development of tomographic luminescent imaging instruments that can generate 3D images promise to enhance both sensitivity and spatial resolution of the T cell signal using this model, and should permit finer mapping of the real-time dynamics of T cell distribution to all tissue sites in future studies.

## Conclusion

We have characterized a novel transgenic mouse model that utilizes T-cell specific luciferase bioluminescence for tracking T cell homing and migration dynamics *in vivo*. To our knowledge, this represents the first report of real-time, *in vivo *analysis of CD4^+ ^T cell homeostatic proliferation and clonal response to antigen. Although our results are largely confirmatory of prior studies that have examined T cell responses through direct tissue-based analyses of T cell numbers and distribution, implementation of the T-lux model has provided some important new insights, and highlights certain advantages of this approach for tracking the *in vivo *T cell response.

## Abbreviations

CCD: charge-coupled device; T-Lux: T cell-specific luciferase transgenic mouse; hCD2: human CD2; LN: lymph node; ROI: region of interest.

## Competing interests

The authors declare that they have no competing interests.

## Authors' contributions

The authors contributed to the work as following: JC analyzed data and wrote manuscript; KD performed research; TC contributed design and analytical expertise; KZ designed research and contributed to the preparation of the manuscript; and CW designed research, analyzed data and wrote manuscript. All authors read and approved the final version of the manuscript.

## Supplementary Material

Additional file 1 **Early time point Region of Interest (ROI) gating for bioluminescent imaging**. The additional figure contains a detailed gating strategy for quantification of bioluminescent signal arising from specific anatomical sites. Click here for file
