# Supplementary figures and images for: Bioluminescence-based visualization of CD4 T cell dynamics using a T lineage-specific luciferase transgenic model1
Source: BMC Immunol. 2009 Aug 3;10:44. doi: 10.1186/1471-2172-10-44 (PMC2736162; doi:10.1186/1471-2172-10-44)

A.

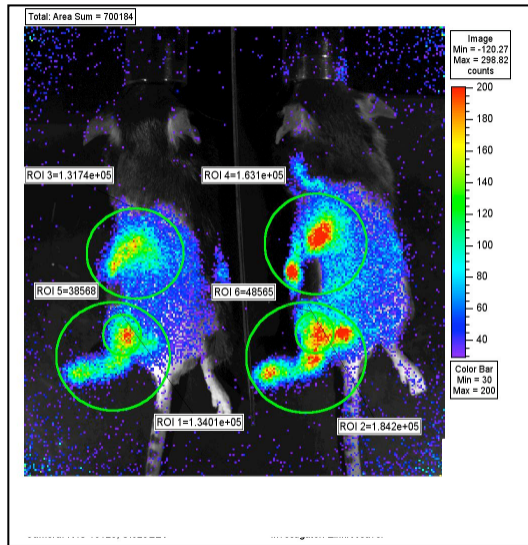

B.

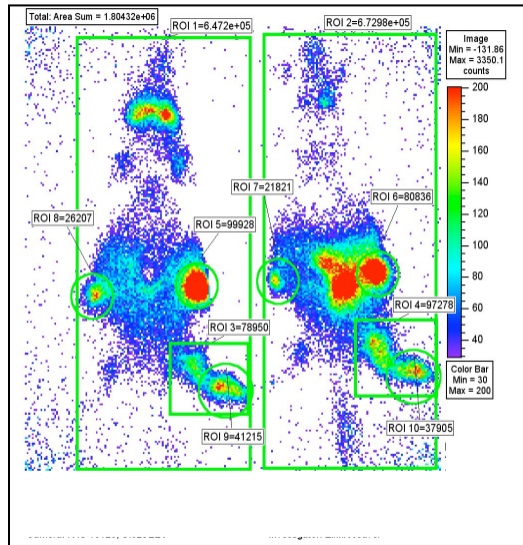

Supplement: Additional file 1 — Early time point Region of Interest (ROI) gating for bioluminescent imaging. The additional figure contains a detailed gating strategy for quantification of bioluminescent signal arising from specific anatomical sites. [file 1471-2172-10-44-S1.pdf]
